# An informatics supported web-based data annotation and query tool to expedite translational research for head and neck malignancies

**DOI:** 10.1186/1471-2407-9-396

**Published:** 2009-11-13

**Authors:** Waqas Amin, Hyunseok P Kang, Ann Marie Egloff, Harpreet Singh, Kerry Trent, Jennifer Ridge-Hetrick, Raja R Seethala, Jennifer Grandis, Anil V Parwani

**Affiliations:** 1 Department of Biomedical Informatics, University of Pittsburgh, Pittsburgh, PA, USA; 2 Department of Pathology, University of Pittsburgh, Pittsburgh, PA, USA; 3 Department of Epidemiology, University of Pittsburgh, Pittsburgh, PA, USA; 4 Department of Otolaryngology, University of Pittsburgh, Pittsburgh, PA, USA; 5 Roswell Park Cancer Institute, Buffalo, NY. USA

## Abstract

**Background:**

The Specialized Program of Research Excellence (SPORE) in Head and Neck Cancer neoplasm virtual biorepository is a bioinformatics-supported system to incorporate data from various clinical, pathological, and molecular systems into a single architecture based on a set of common data elements (CDEs) that provides semantic and syntactic interoperability of data sets.

**Results:**

The various components of this annotation tool include the Development of Common Data Elements (CDEs) that are derived from College of American Pathologists (CAP) Checklist and North American Association of Central Cancer Registries (NAACR) standards. The Data Entry Tool is a portable and flexible Oracle-based data entry device, which is an easily mastered web-based tool. The Data Query Tool helps investigators and researchers to search de-identified information within the warehouse/resource through a "point and click" interface, thus enabling only the selected data elements to be essentially copied into a data mart using a multi dimensional model from the warehouse's relational structure.

The SPORE Head and Neck Neoplasm Database contains multimodal datasets that are accessible to investigators via an easy to use query tool. The database currently holds 6553 cases and 10607 tumor accessions. Among these, there are 965 metastatic, 4227 primary, 1369 recurrent, and 483 new primary cases. The data disclosure is strictly regulated by user's authorization.

**Conclusion:**

The SPORE Head and Neck Neoplasm Virtual Biorepository is a robust translational biomedical informatics tool that can facilitate basic science, clinical, and translational research. The Data Query Tool acts as a central source providing a mechanism for researchers to efficiently find clinically annotated datasets and biospecimens that are relevant to their research areas. The tool protects patient privacy by revealing only de-identified data in accordance with regulations and approvals of the IRB and scientific review committee.

## Background

Due to advances in cancer diagnostics and therapeutics over the past few decades, the complexity of these areas has increased and the treatment and management of cancer patients has become even more of a challenge. Growth in translational research, focused on the identification and validation of disease biomarkers, has led to the development of biorepositories that are capable of providing high quality biospecimens with detailed clinical annotations. Recent developments in information technology have been an important enabling factor in this process by providing the basis for an informatics architecture that can carry out and harmonize many of the functions that biorepositories need to perform. Informatics as a discipline involves the collection, classification, storage, retrieval and dissemination of recorded knowledge [1]. Over the last decade, the development of tissue banking and informatics systems has been recognized as crucial to the pursuit of detailed translational cancer research. However, it also has the potential to truly transform systems: the basic observation of the information age is that the sharing and dissemination of knowledge does not diminish its value, but can actually create new synergies and applications of that knowledge by increasing its utilization and accelerating the rate of advances. In order to truly take these systems into the information age, semantic and syntactic interoperability across multiple institutions must be achieved, which requires a robust system of clinical annotation based on controlled vocabulary and ontology [2]. The recommendation in the RAND Corporation's Case Study of Existing Human Tissue repositories, "...the collection of consistent and high quality data associated with every biospecimen and employing a standardized set of common data element for annotation..." is now broadly considered best practice: this general consensus reflects the need for such standardization [3-5]

The head and neck cancer virtual biorepository is funded through a Specialized Program of Research Excellence (SPORE) grant (P50) mechanism, which promotes interdisciplinary research involving both cancer patients and populations at risk for cancer. SPORE investigators work collaboratively to plan, design and implement research programs that may impact cancer prevention, detection, diagnosis, and treatment. Additionally, the SPORE program supports collaborative efforts within the individual multidisciplinary SPORE teams, inter-SPORE collaborations, partnerships with other NCI/NIH programs, and public-private partnerships with industry and non-profit organizations. Important characteristics of the program are the inclusion of patient advocates in SPORE activities and international cooperation with investigators in Europe, Canada, Asia, and Mexico. SPOREs in Head and Neck (H&N) cancer support translational research on cancers of the upper aerodigestive tract and thyroid cancer. The SPORE head and neck cancers program was established in the year 2001, with three new SPORE programs established and funded in the year 2002, the fourth in 2004, and the 5^th ^in 2007.

In this manuscript we will describe the contribution of the Department of Biomedical Informatics at University of Pittsburgh in the development and implementation of a bioinformatics infrastructure developed on a set of common data elements to integrate data from various clinical, pathologic and molecular resources into one design in order to support basic science and clinical as well as translational research. The use of common data elements (CDE) provides semantic and syntactic interoperability across multiple hospital data resources. Common Data Element sets were developed by mutual consensus among professionals using the North American Association of Central Cancer Registries (NAACR) [6] standards and College of American Pathologists (CAP) protocol and checklist [7].

The virtual biorepository provides web-based data entry and data query tools. The clinical annotation warehouse system is supported in a Java-based three-tiered architecture. It also provides image storage and analysis tools, in addition to serving as a data warehouse for clinicopathological and follow-up datasets. The resource allows investigators to utilize the warehouse to obtain high quality clinically annotated biospecimens for clinical and translational cancer research. The query tool accesses the central database through a highly constrained "click and point" interface. The resource will have no access at any time to patient identified data and tissue will only be made available to researchers upon user authorization. Furthermore, by integrating multimodal datasets in the annotated tissue repository, and making this information available through the creation of query and visualization techniques for these diverse data types, this provides better characterized tissues for research and allows users to select them with a greater degree of flexibility.

## Methods

### Standard for Subject Enrollment/Exclusion

All participants enrolled in the SPORE Head and Neck Neoplasm Project (Epidemiology of Genetic Susceptibility to Head and Neck Cancer) are consented and enrollment criteria are based on following inclusion/exclusion protocols:

### Case definition

The inclusion and exclusion criteria, used to define the case series, appear below.

#### Inclusion Standards (case series)

I. Age 18-79 years on date of first diagnosis of qualifying head and neck cancer. Biopsy proven primary squamous cell cancer at a head and neck site. For the purposes of this research, head and neck cancer sites include primary tumors coding to Chapter 3 (Lip and Oral Cavity), Chapter 4 (Pharynx, including base of tongue, soft palate, and uvula), Chapter 5 (Larynx), or Chapter 6 (Nasal Cavity and Paranasal Sinuses) of the American Joint Committee on Cancer (AJCC) Staging Manual (6th edition) [8].

II. Study enrollment based on acquisition of informed consent and collection of questionnaire data at the time of diagnosis or after one year from the initial diagnosis of the qualifying head and neck cancer.

#### Exclusion criteria (case series)

I. Age less than 18 years on date of first diagnosis of qualifying head and neck cancer.

II. Age more than 79 years on date of first diagnosis of qualifying head and neck cancer.

III. Absence of a clinical pathology report documenting invasive cancer involving a head and neck site.

IV. Histopathologic diagnosis other than squamous cell carcinoma.

V. More than one year elapsed time since date of first biopsy diagnosis of most recent primary squamous cell carcinoma at a head and neck cancer site.

Diagnoses are subject to verification by a pathologist. The investigators will remove a participant from this research study if the final pathology report does not confirm a provisional diagnosis used for study enrollment purposes. If the final pathology report does not demonstrate primary squamous cell carcinoma of the head and neck, the investigators will remove the participant from the study and his/her data and blood, urine, and saliva sample will be rendered anonymous and destroyed.

### Control definition

The inclusion and exclusion criteria, used to define the control series, appear below.

#### Inclusion criteria (control series)

Age 18-80 years on date of enrollment.

No personal history of cancer at a head and neck site (based on eligibility screening interview and/or review of ENT or Dental Clinic medical record).

I. (If a clinic control) Clinical examination (by personal ENT physician or dentist), without clinical suspicion of head and neck cancer, based on testimony of clinician or review of primary medical records.

#### Exclusion standards (control series)

I. Age less than 18 years on date of enrollment.

II. Age more than 80 years on date of enrollment.

III. Subject self-report of personal history of cancer at a head and neck cancer site.

IV. (If a clinic control) Indication in ENT or dental clinic record of personal history of cancer at a head and neck cancer site.

V. (If a clinic control) Physical findings, on head and neck clinical examination, that creates a suspicion of cancer at a head and neck cancer site.

### SPORE Head and Neck: Tissue Bank

#### Eligibility standards

I. Participants under the care of doctors in the Department of Otolaryngology at the University of Pittsburgh, as well as healthy controls who are not under the care of our doctors will be accrued.

II. Participants who are at risk for or have developed primary, recurrent, metastatic or second primary cancer of the head and neck will be enrolled. In addition, people with a non-cancerous diagnosis, who may or may not be having surgery, will be asked to participate as controls.

III. Any age 18 years or older. Children are not included since the incidence of head and neck cancer in individuals under the age of 18 is remarkably rare.

IV. Written and informed consent will be obtained. Mentally-impaired adults or those are not capable of understanding the consent information will not be included in this research project.

### Standards for Data Collection and De-identification

#### Development of Common Data elements (CDE)

Common data elements are developed to facilitate annotation of cases at epidemiology, demographics, clinicopathology and follow-up status. CDEs also provide characterization of biospecimens collected at University of Pittsburgh Medical Center (UPMC). The CDEs encompass cancer registry data (treatment, vital status, recurrence etc) at the case level. CDEs provide specimen level pathology data elements to describe tumor staging grade and histological type, along with genotype data elements and block level annotation. The CDEs for standardized data collection were developed from several sources, including the North American Association of Central Cancer Registries (NAACCR)[6] and College of American Pathologists (CAP) [7] cancer protocol and checklist, and the Association of Directors of Anatomic and Surgical Pathology (ADASP)[9] and American Joint Committee on Cancer (AJCC) [8] cancer staging manuals. This was achieved through the combined efforts of domain experts from various specialities using core standards under the supervision of the SPORE Head and Neck Neoplasm Biorepository coordinating committee. The CDEs developed are ISO/IEC 11179 (International Standards Organization/the International Electrotechnical Commission) compliant. CDEs define a number of fields and relationships for metadata registries, including a metadata model for defining and registering items, of which the primary component is a data element [10].

The informatics supported architecture allows the CDEs that are used to annotate the biospecimens to be semantically and syntactically interoperable by describing them in the form of metadata or data descriptors. The individual CDEs are associated with an attribute of an object or concept, and valid value or values. For example, "patient age at diagnosis" is the CDE that consists of "patient" as an object, "age at diagnosis" as an attribute and the representation value domain in "years". To collect data based on approved CDEs, the data managers need to know the fundamental definition of the data element, standards of data collection, mutually accepted values or codes for the data element, and the acceptable data format for inclusion in the central database. Although the concept of formalized metadata is fairly straightforward, it has rarely been taken into account by clinical and research groups building databases [11].

#### Data Collection, Development of Data Collection Application and Data Transmission

The research nurse coordinator is responsible to consent the patient at the time of physician office visit before the surgery or undergoing surgical procedures. At the time of consent self-administered questionnaires are handed over to the patient to collect information on demographics, risk factor exposures including tobacco and alcohol use, sexual behavior and previous medical history. Structured pathology data is retrieved from the coPath system plus application (Version 3.0.2.74 Cerner DHT, Inc.) in the form of synoptic reports. The surgical pathology report and all histological sections available to the head and neck pathologist are reviewed to correctly categorize each case. The pathologist then selects representative slides and paraffin blocks according to a study standardized protocol. The selected slides show specific features of the case likely to be of interest for scientific investigators. After the pathological data are reviewed, the certified tumor registrars review and extract clinical and follow-up data. The data are collected through the cancer registry information system (CRIS) or manually from the patient medical charts. Collected data are then stored by using common web-based data entry forms that are correlated with the CDEs developed within the head and neck neoplasm organ specific database.

#### De-identification Process and Honest Broker Concept

The SPORE in head and neck cancer virtual biorepository is structured to protect patient privacy and confidentiality according to Institutional Review Board (IRB) regulations and HIPAA (Health Insurance Portability and Accountability Act) approved protocols. The database discloses only deidentified patient information and displays no links to patient identifiers (name, date of birth, procedure date, therapy date, etc). The only linkage is kept within the institution and the database generates de-identified dataset upon query by the end users (the so-called "safe harbor" approach to HIPAA compliance) [12]. The "safe-harbor" approach involves exclusion of all 18 identifiers enumerated in section 164.514(b) (2) of the regulations. Thus for example, a participant's age is presented as age range, rather than the date of birth, and therapy dates are provided in months from first positive tissue diagnosis to therapy start date rather than presenting a precise calendar date. These are some of the measures adopted to protect the identity of patients while still providing sufficient information for research purposes. All data requests are tracked in the secure SPORE Head and Neck Neoplasm Database regardless of whether the purpose is clinical or research related.

The de-identification process is performed by an honest broker, which acts as a filter between completely identified confidential clinical patient information and the completely de-identified data made available to the research community. An honest broker is an individual, organization or system acting on behalf of the covered entity to collect and provide health information to the investigators in such a manner whereby it would not be reasonably possible for the investigators or others to identify the corresponding patients/subjects directly or indirectly. The honest broker cannot be one of the investigators or researchers. A researcher may use the services of an honest broker service to obtain the Protected Health Information in a de-identified manner. The honest broker service will de-identify medical record information by automated or manual methods. All honest broker services are approved in advance by both the IRB of record and University of Pittsburgh Medical Center (UPMC). If an honest broker service is not part of the UPMC covered entity, a valid business associate agreement with UPMC is executed with UPMC in order to access UPMC-held Protected Health Information for de-identification. If an honest broker service is to be used to obtain de-identified Protected Health Information, this fact must be identified in the study's IRB submission. The honest brokers in this case are individuals who have clinical responsibilities as tissue bankers in the Health Sciences Tissue Bank (HSTB), postdoctoral fellows who manage the pathology data or cancer registry specialists in the UPMC Network Cancer Registry. Based on their clinical job duties, their educational backgrounds and experiences vary. Depending on the nature of the projects, these bankers can work autonomously or collaboratively to meet biospecimen and data needs [13].

### Accuracy Assessment of Multimodal Datasets

The SPORE Head and Neck Neoplasm Database has collected essential information, such as patient demographic, pathology, treatment, recurrence and risk factor exposure data, from head and neck neoplasm patients at the University of Pittsburgh Medical Center since 1980. After importing the multimodal data into the head and neck organ specific database, accuracy is assessed by trained and certified personnel, using policies, variable constraints, and logical tests established by the resource. Data are collected by the data managers from electronic sources including the Medical Registry System (MRS), pathology reports from coPath Plus and the social security death index for entry into the database. The evaluation of the collected data is done using the following approach.

The first step is to evaluate discrepancies between the database quality check curators. The primary focus of data accuracy assessment is on tumor record, staging, histology, diagnosis, treatment, recurrences and risk factor exposure data. The selected data fields are categorized on separate error rate for primary, secondary and tertiary priority fields. The error rate for each case is calculated depending upon number of discrepant entries and the number of fields evaluated for a case. Evaluated number of fields and number of discrepant entries for selected cases are used to find the error rate for each discrete priority level data field.

The second step evaluates the accuracy of database entries by comparing them to the electronic data source from which data are collected. Data fields have been divided into high priority, secondary priority, and tertiary priority. For those fields listed as either high priority or secondary priority, the number of deviations from the entry per total number of high priority fields assessed will yield an estimate of the error rate for each priority tier. For high priority fields, which include patient demographic and clinical data and tumor pathologic data, the estimated error rate should not exceed 2%. For secondary priority fields, which include risk factor data, there is a slighter higher allowed rate of 5%. The high priority fields are composed largely of fields extracted from the UPCI tumor registry. The UPCI tumor registry guidelines require that the error rate not exceed 3%. A threshold of 2% was selected for high priority fields as a compromise between a desire for a research quality database with an error rate less than 1% and the practicality of the resources and effort already required to meet the mandated less than 3% error rate guideline. A less than 5% error rate was estimated to provide reasonable quality risk factor exposure data, which have been collected using instruments that have varied to some degree over the time period of collecting of these data. An error rate will also be calculated for tertiary fields; however, no threshold has been set for the tertiary field error rate to initiate a database update. Initially, 1% of the subjects are evaluated. If discrepancies are within error rate guidelines for primary and secondary fields, a further 5% of subjects will be randomly selected using the same strategy, and estimated database error rates will be calculated separately for the first and second priority fields for the 5% sample. If the error rate guidelines are not met for the 1% initial evaluation, a careful analysis of the differences will be performed and discrepancies identified. A quality check of all database subjects will be performed, focusing on discrepant fields. After the quality check has been completed, a second sampling of 1% of subjects will be performed. This sampling will exclude subjects sampled in the prior evaluation. Error rates will be determined, and if error rate guidelines are met, a further 5% of subjects will be evaluated using the same criteria.

The third step involves comparing the data in the database to data in primary sources such as clinical charts and pathology reports. Subject sampling will be performed and data field error rates will be calculated as above. However, due to the amount of time and effort required to review primary records, only 1% of database subjects will be evaluated. In order to best identify differences in electronic versus primary data, these 1% of subjects will be a randomly selected subset of the 5% of subjects assessed for consistency with the electronic database.

### Accuracy Assessment of pathological data

The pathology data pertaining to each case of paraffin-embedded tissue block e is entered after complete assessment by trained head and neck pathologists. This is an independent review process in which a series of randomly selected cases are re-reviewed. The data managers randomly select cases for independent review from those added to the head and neck neoplasm database within certain cut-off dates. The independent review material consists of 2 to 5 pathologic matrix slides, defined as slides selected for annotation, for each case. Once the pathologist receives the slides, he/she will evaluate and annotate the matrix slides and histology CDE data for the case using their established processes. The pathologist will then scrutinize the histology CDE data for observer variability and diagnostic error rates. This process occurs biannually and is established to check specimen resource quality. Any discrepancies identified through independent review are communicated to the pathology subcommittee. The pathology subcommittee then discusses these findings in the subsequent general meeting of the coordinating committee through a formal report with recommendations for changes in process as indicated by the independent review findings. Any errors discovered during the independent review process are corrected [14].

### SPORE Head & Neck Neoplasm Database

#### Integrated Informatics Modal

The overall system is designed as a multi-tiered application using Oracle 10 g Database Server, Oracle 10 g Application Server, and MOD_PLSQL, also known as PL/SQL Server, on a Compaq DL360 Server running Win2K with SP. This application utilizes the Oracle HTTP Server and MOD_PLSQL extensions to generate dynamic pages from the database to the users. The database is Oracle 9.2.0.1 Enterprise Edition implemented on a SunFire V880 Server running Solaris 2.8. Approximately four months period of time was spent in the development and deployment of the initial application set up. Application enhancement and expansion are continuous process that is carried out based upon end user requirements.

The informatics model depicts the SPORE Head and Neck Neoplasm Database in the following layers: *Schema layer *-this consists of concrete data and data relations. All classified data is stored as numbers and keys. *Metadata layer *- demonstrates data in terms of data elements and "groups of data elements". Data descriptions such as data attributes, display attributes, valid values, DB Link, validation rules and documentation are supported in metadata. The Metadata layer defines the application layer. *Procedures/function layer *- depicts a set of dynamic functions (in PL/SQL or Java) with control data transformation at the back end. The procedures accommodate changes in the metadata and immediately reflect the changes in the application layer. *Application layer (Form builder) *- presents a set of "applications" including the metadata dictionary builder and manager, user management, data entry/transfer, query, display, etc. The appearance may differ depending on user privileges. These differences are driven by the metadata and user management.

#### SPORE Head and Neck Neoplasm Database Model

**Figure 1:**
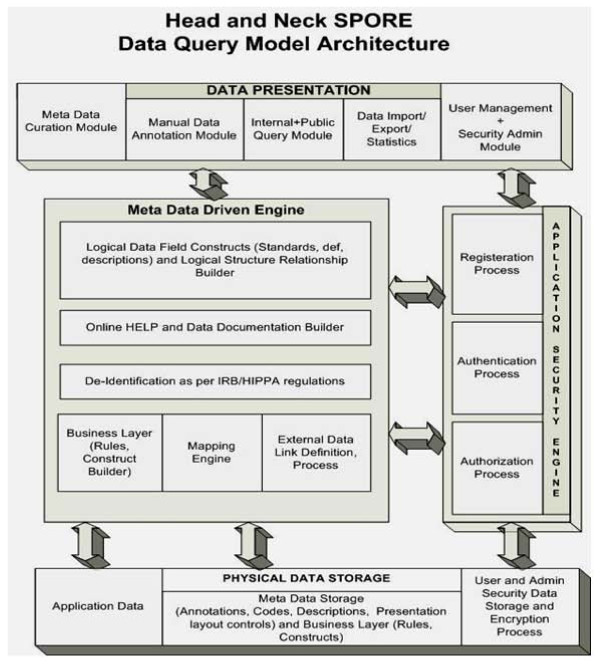
**Presents the actual data model architecture for Organ Specific data warehouse implementation for head and Neck SPORE program**. This architecture supports data query and data entry and data de-identification and *user *management module.

The SPORE Head and Neck Neoplasm Database consists of the following integrated application layers that maintain data query/entry, data de-identification and the user management module (Figure 1).

#### i. Presentation Layer

**Figure 2:**
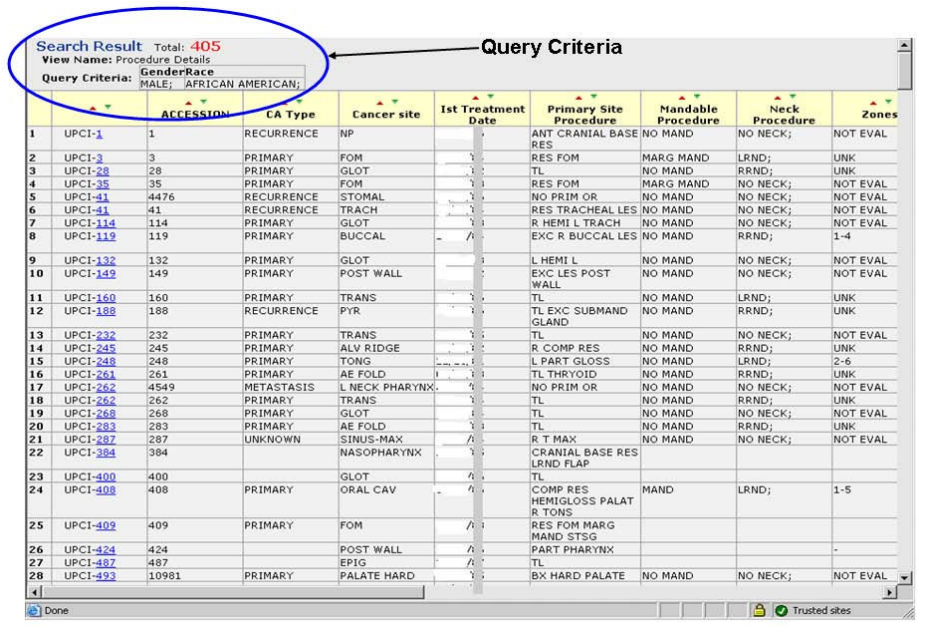
**Presents the ability to export data query results to Microsoft Excel file format along with query criteria for future analysis and allow users to sort on every data field**. Exported data can be deidentified or identified based on use access authentication.

This contains the following components: *metadata curation *is used by data administrators and CDE curators for registering new CDEs or editing definition of existing CDEs. The *administrator security system *is used by the application administrators to grant, revoke or limit privileges to new and existing users. *Manual annotation *is used by honest brokers or domain experts for collecting information regarding patients registered for the study. *Data query *is used by the honest brokers and research community to run criteria based queries. The query results show identified and de-identified outputs depending on the individual roles and privileges granted by application administrators. This tool provides two levels of access to researchers who participate in the SPORE Head and Neck neoplasm study. The first level of access is to have broker view of the consented patients for their own study and second is a de-identified view on all the patients for other studies for which they do not have access but want to study and analyze overall trends. The *data import/export *component provides users an option to electronically import preformatted data from existing systems or export data for their desktop analysis (Figure 2).

#### ii. Metadata Engine

**Figure 3:**
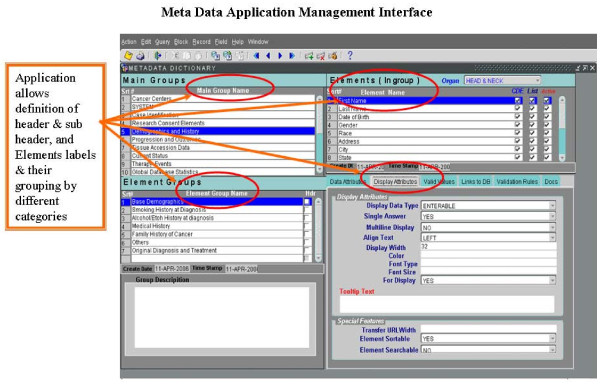
**Presents the Meta Data Application management interface for developing the metadata common data elements, data descriptions, data presentation and help documentation building and mapping of dictionary CDE definitions to physical data tables by Application Data Manager and System Designer**.

The Metadata Engine is based on the development of *Common Data Elements *that are used to hold application data structure for data elements/fields as defined by the SPORE Head and Neck Neoplasm Project working group. The *HELP builder *is used for each data element/field with its detailed definition of business rules and usage. The *business rules engine *constitutes business rules for how multiple elements can be combined with simple numerical and algorithmic techniques to report complex values for decision support and statistical time sensitive outputs. The *mapping engine maps *logical and physical layers of design that facilitate data retrieval and storage (Figure 3).

#### iii. Security Engine

The security engine secures the application at three levels: the first is *registration *of new user accounts and requesting application roles. Second is *authentication *byadding/editing user information, and lastly, *authorization *is granting or revoking user roles and privileges.

#### iv. Physical Data

The physical database tables are presented in the data warehouse in a three step fashion. First is the *application database *that holds case data contents in a metadata coded format. Second is the *metadata database*, which holds metadata definitions and descriptions for all the attributes and values in the system. The third one is a *security database *which drives the security and authorizations definitions and assignments.

## Results

The Head and Neck Neoplasm Database contain over 6553 archived and prospective head and neck neoplasm cases. Biospecimens that are collected from over 10607 surgical resections and biopsies, as well as whole blood and DNA samples, are included in the resource. The majority of these cases consist of archival paraffin blocks from surgically treated patients.

The SPORE Head and Neck Neoplasm common data elements (CDE) are developed using previously described standards (NAACCR, ADASP & CAP) for building CDE datasets. A multi-tiered application using Oracle 10 g is the platform for the web based query tool. Access to the database is available only to investigators approved by the IRB and Scientific Review Committee. It permits users to query the data related to the specimen that they have received from the resource for their approved studies and enables them to develop their own case lists. The query results display only de-identified datasets linked to each case via pre-defined views.

### Work flow for entering the data into the database using web based data entry tool

**Figure 4:**
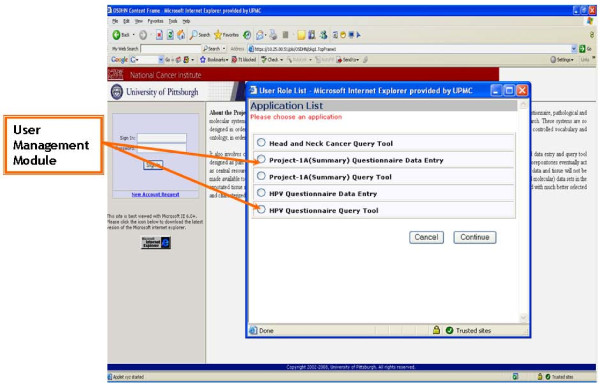
**Presents the User management module for controlling the user access to different applications modules within the data warehouse by Application Data Manager and System Administrator**.

The datasets collected from different sources are manually entered in the databases by utilizing the data entry tool. This tool is password protected and controlled by a user authentication process/manager controlled user management module (Figure 4). The local (physical) tissue bank identifies cases appropriate for inclusion in the Head and Neck Neoplasm Virtual Bio-repository. All cases entered into the virtual biorepository are based on interview data and the complete associated tissue/biopsy data for the participant. The local tissue bank pre-processes data on these cases. The most important component of pre-processing is de-identification. All de-identification occurs at the local banks. No identifiable data is sent to the virtual biorepository. De-identified data are entered into the warehouse through a web site. The data entry web site uses radio buttons, combo boxes and other highly constrained data elements. The local banks label each case with a de-identified number. This number is used to link the information in the warehouse to the cases in the local banks. The linkage codes are stored locally, using appropriate electronic and physical safety measures. Only the local banks have access to these linkage codes. The warehouse contains very minimal demographic data and complies with all HIPAA requirements. Cases entered into the virtual biorepository are scanned for logical errors (e.g.: first recurrence before diagnosis etc.).

### Workflow for querying the database

**Figure 5:**
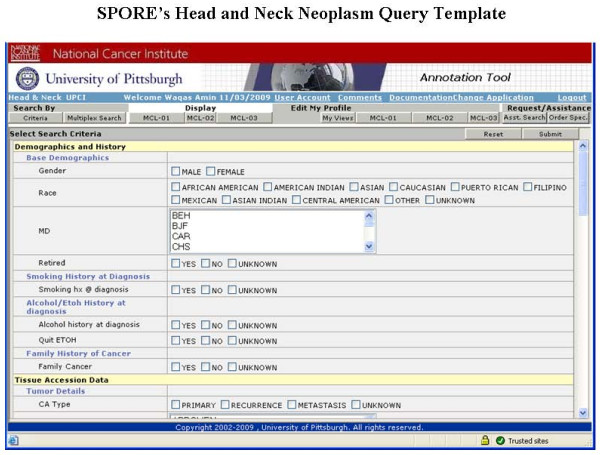
**Presents the metadata driven search query template for the end user to define and build their data query criteria and get the query based results**. In addition multiplex query tab allows to do free text search.

**Figure 6:**
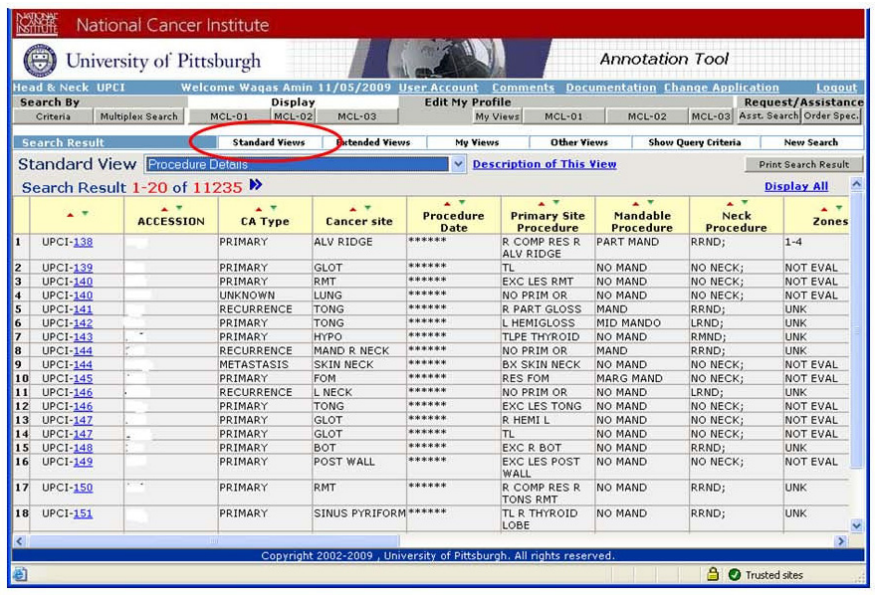
**Presents Standard data views defined by System Administrator and based upon user selected query criteria; User's have the ability to define their own data views and data cohorts**.

**Figure 7:**
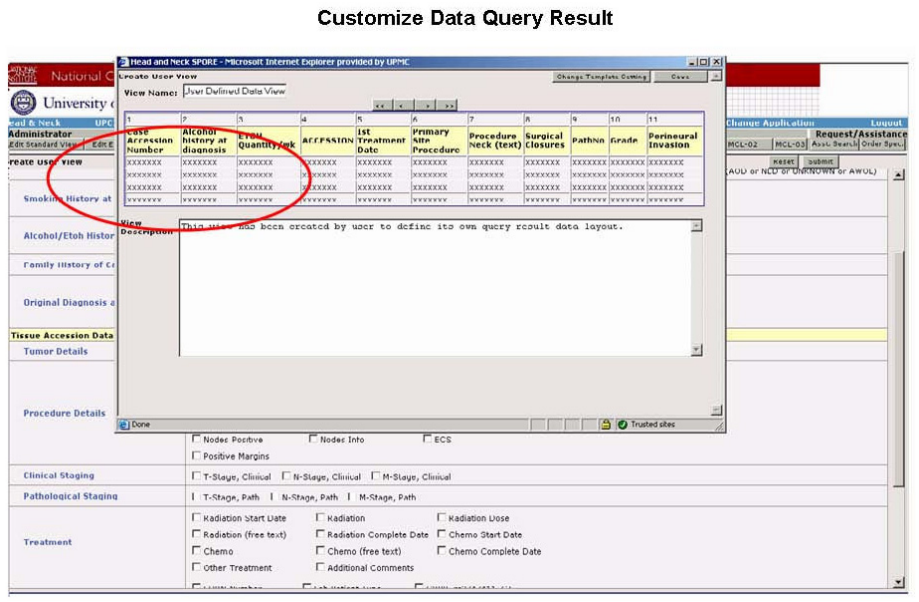
**Presents the user interface with ability to customize data query result page view**.

Initially, access is limited to members of the SPORE Head and Neck Neoplasm working group using a user name and password system. The data administrator can provide a user name and password for approved researchers or nursing coordinators to access the tool. The query tool access to the central database is through a highly structured "click and point" interface. The data in the database allows queries on approved data elements and also is based on the researcher's IRB approval. The specificity of the data returned depends on the user's profile. There are four user profiles;

**i. Approved Investigator Query tool **is a password-protected application which is distributed to those research investigators who have approved research protocols within the SPORE Head and Neck Working Group. It allows users to refine and compile case lists for their application and also to mine and modify the data set on the cases where they have received biospecimens. The query tool provides search on all the annotated data associated with each subject through multiple pre-defined standard views of the data set and also allows users to customize their own views and save them under their account (Figures 5, 6 and 7).

**ii. Data administrator Query tool **is a password-protected tool available only for the internal data administrators. It is meant to be used by data administrators to address quality assurance issues regarding the data. The main difference between this and the approved investigator tool is that this tool allows the user to search by "all Subjects" or "limited Subjects" based on consent for a particular study.

**Figure 8:**
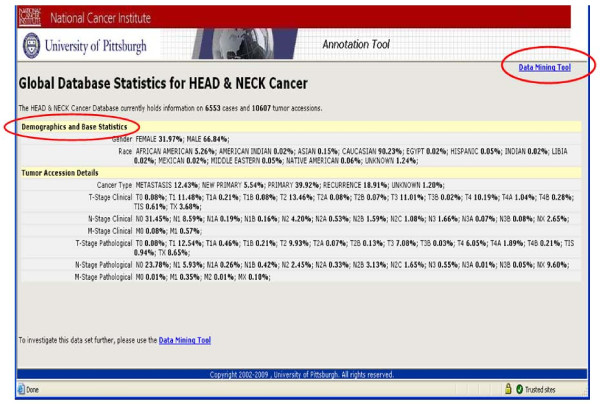
**Presents the overall statistics for the Head and neck SPORE Data repository and these statistics can be specific to individual queries**.

**Figure 9:**
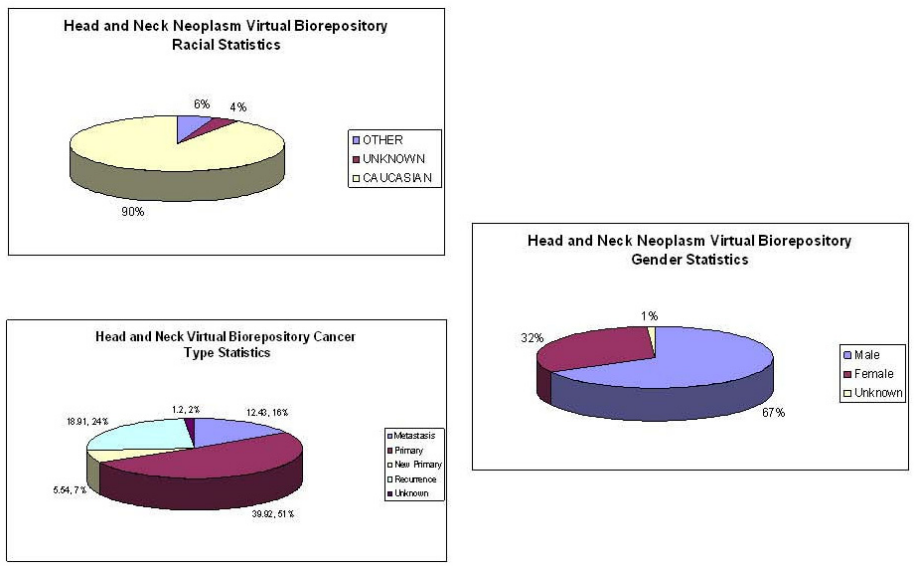
**Presents statistics on gender, race and cancer type on all head and neck neoplasm cases**.

**iii. Public Query tool **is available to the general public and accessible through the main home page. The output display of a public query will be the accrued number of cases, specimens in the database that meet the criteria of the query and general statistics on a limited number of data elements. It is designed to allow interested investigators to determine if the resource will be applicable to their research needs (Figure 8 &9).

iv. Human Papilloma Virus (HPV) questionnaire and Project-1 Epidemiological data:

The Project-1 interviewer-administered and HPV self-administered study questionnaires captures patient epidemiological and sexual behavior information. The annotation tool provides manual data entry and query on de-identified information obtained from these questionnaires through a "point and click" interface that internally integrates with annotated clinicopathology data sources.

### Integration of Multimodal Data Sources

The SPORE Head and Neck Neoplasm Database supports integration with other prominent sources of patient related information regarding diagnosis, follow-up, treatment, as well as questionnaire data. The pathology annotation and inventory data of banked tissues are tracked by the biorepository team by utilizing synoptic reports from the anatomic pathology information system. The research registrar and data manager gather follow-up and outcomes related data by reviewing medical records. The questionnaire data provides patient epidemiological and sexual behavior information. This allows the users of this biorepository to query the complete case and obtain an overall view of the patient's progress and history.

## Discussion

The growing importance of translational research and development of informatics tools capable of analyzing clinical tissue samples has increased the SPORE research community demand for high quality and well annotated biospecimens. To satisfy this demand, the SPORE Head and Neck Neoplasm working group has established an integrated head and neck neoplasm biobank and web-based database query tool. This system is built on an underlying architecture of common data elements for characterization of tissue samples and clinical follow-up data, supported by an essential quality assurance process. In the development of the SPORE Head and Neck Neoplasm Database, experts from various specialized fields invested a significant amount of time and effort to design and deploy a high quality and well annotated biospecimen resource. In the clinician's domain, the focus was on creating CDEs that aim at the acquisition of information adhering to current standards on clinicopathology, follow up, and cancer outcome. Special consideration in this regard was given to a future extrapolation of at least five years during which any data may become clinically significant. Data from patient questionnaires was also included in CDE development. The resultant data sets are expected to satisfy the needs of the SPORE research community and augment future translational research activities. The cancer registries, bioinformaticians and research nurse coordinators who are responsible for data gathering provided invaluable input regarding metadata and data types in the process of CDE development.

It is imperative that CDEs and the metadata coupled must be defined with absolute clarity and made comprehensible to the people involved in data collection. The aim of collecting high quality annotated data was realized through the implementation of ISO 11179 compliance standards [15]. Despite the fact that it is a rather simple, clear cut concept, the formalized metadata model has seldom been implemented by research and clinical groups in the process of data base development. Technical issues pertaining to CDEs were resolved by the database developers and informaticians who also designed the architecture for CDE deployment.

This also allows a much closer monitoring of trends and issues in cancer, which is essential to the ultimate goal of the NCI, the lessening of the burden of pain and suffering from cancer [16]. The NIH and NCI strategic roadmaps clearly define the value of tissue banks and informatics in supporting these objectives [17].

### SPORE head and Neck Neoplasm Database Annotation Interface

In the annotation process a data entry web interface is used for manual entry of all relevant clinical, cancer registry and pathologic information. Strict protocols are maintained for integration, de-identification and standardization during data entry. The integration process involves collecting prospective data and incorporating retrospective data from multiple clinical systems over time regarding a selected patient. For example, data must be gathered from different sources such as the pathology information system, medical archive system, tissue bank inventory system and the cancer registry.

#### Annotation Challenges

There are two major obstacles in this process. First is the correct identification of a certain patient in multiple systems or hospitals and the second is pinpointing only applicable information. In order to track follow-up data on patients who utilize multiple health care systems, one must be able to incorporate the patient data from various sources. This can be achieved by common linking patient identifiers or health information; nevertheless discrepancies can occur owing to errors based on data entry issues or the absence of specific identifiers unique to the patients. The head and neck neoplasm virtual biorepository has applied a solution to address these obstacles which is mainly a process of manual data collection and entry by the data managers. This ensures that the focus on data quality is maintained. However the collected data that has temporal relationship still requires to be evaluated for context before it can be captured into a database. The approach was carried out by the resource to tackle data context that is basically a manual process of data collection and entry. Furthermore this method allows incorporation of more data elements than needed for the resource, including data elements not relevant to head and neck neoplasm virtual biorepository. These additional data elements provide integration with other data sources and verified data integrity. We hope to overcome such challenges in the future using automated data retrieval through electronic queries of existing systems (i.e. MARS and the Pathology Information system). These would require the use of messaging standards (i.e. HL7 and DICOM), and vocabulary standards (i.e. SNOMED and UMLS). Despite the effectiveness of these measures the process may be hampered by the presence of unstructured systems using free text in lieu of structured data fields that complicates searching for cases within these databases. In this scenario manual annotation may be utilized to resolve this issue.

### SPORE head and Neck Neoplasm Database Interface

The query tool was designed as a layered web based tool using an Oracle database platform to share data between internal members as well as researchers approved by the IRB and Scientific Review Committee. In addition to its security features, this query tool enhances the expansion capabilities for incorporating new data elements or integrating existing system features. Emphasis has been placed on the ability to define user access level to the datasets, and we also provide functionality for data monitoring. The Head and Neck SPORE query tool has the different modules to represent the data in different ways for different levels of access.

The public query tool presents part of the data as a public view because it's available for sharing across the domain experts. The query tool security layer automatically reviews the user rights based upon its login information. Depending upon the user access rights the user's are allowed to view only that part of the data for which principle investigator or system administrators have allowed access to a particular user. Normally public users are not allowed to see the actual patient's identifiers' according to HIPAA rules and regulations. The part of the data is not even accessed from database tables if users are not authorized to see and system masked data fields to the presentation layer. The public users can still customize their own data views for export/import purposes but cannot customize or change the Standard Views or Extended views (Shared by all levels of users). The standard view displays the data on subset of collected data like demographic, epidemiology tumor accession etc. The extended view displays the entire data sets collected on all data fields.

In approved investigator query view the users can act as both Honest Broker and researcher depending upon IRB consent. In most of these cases IRB-approved researchers have consent to see patient data for their patients enrolled in their studies and have honest broker view for data on these patients. For all other patients the view is masked as explained earlier in the manuscript. These users can customize their own views but can not change/create/delete the Standard or Extended views of data sets.

### Comparison with other Databases Developed Using the Identical Technology

The SPORE's Head and Neck Neoplasm Virtual Biorepository is built on the same technology that is used for CPCTR and PCABC [18,19], except that an updated version of software is incorporated as described in this report. The data entry in head and neck neoplasm virtual biorepository is carried out both by manually utilizing data entry tool and electronically through excel and XML files, which is in similar to PCABC but in contrast to CPCTR [20]. The Head and Neck Neoplasm Virtual Biorepository access and utility is currently limited to University of Pittsburgh Medical Center researchers who are participating in the SPORE's head and neck neoplasm project. The SPORE Head and Neck Neoplasm database has been designed around the Oracle database and most of the capabilities of Oracle database server and Oracle application server have been extensively used. The underlying idea is to improve the data storage and retrieval process. This tool is generic in nature and its framework can be used to deploy it for any other disease specific application since it's architecture is based on three tier data structure (Physical Data Layer, Meta Data Layer and Presentation Layer as discussed earlier in this report).

The Physical data layer and Presentation layer or Web interface codes are all written in PL/SQL server pages and java scripts to generate dynamic queries and present them to the front end dynamically generated HTML pages. The middle layer 'Meta Data Layer' is very much independent and can be replaced to implement the same structure for any other organs or disease-specific database application with similar capabilities, without any changes in underwritten code. This tool is not designed to use technology like open source/open access but may still be easily deployed at different sites by having the oracle environment setup and import the oracle schema framework copy. The user is anticipated to be able employ this tool without major technical assistance by following provided instruction documents. Currently, the Head and Neck Neoplasm SPORE application tool is being used internally for UPMC/UPITT users and has not yet been made available to outside researchers. However, it could be easily deployed anywhere on windows or UNIX environment platforms for oracle database 10 g.

The SPORE Head and Neck Neoplasm Database provides query and data entry interfaces for Project-1 (Epidemiological) and Human Papilloma Virus (HPV) questionnaires and genomic/proteomic data. The database provides study base access upon user authentication. The database also provides an inbuilt de-identification option that is controlled by internal routines and coding mechanisms to mask the data as per HIPPA compliance and provides access to users based upon their access level and user group rights. This mechanism has proved to be most efficient means of data de-identifications.

The administrator or principle investigator (PI) access to the Head and Neck Neoplasm Database can have both honest broker and researcher views with the ability to view the most of the data fields. The administer or PI has the ability to create/change/delete/customize Standard and Extended data views in addition to their own views and may even change/revoke other user's access rights in their own group. These options provide both flexibility and control. This tool was developed in-house and has been specially designed to support research community needs not currently met by commercially available tools.

## Conclusion

There have been a mounting number of national and statewide initiatives aimed at supporting the development of tissue banks promoting the sharing of tissue and data resources. At present, multiple institute participation is a feature of several tissue banks including the Cooperative Prostate Cancer Tissue Resource (CPCTR) [21], Pennsylvania Cancer Alliance for Bioinformatics Consortium (PCABC) [18], Cooperative Breast Cancer Tissue Resource (CBCTR) [22], Cooperative Human Tissue Network (CHTN) [23], Cancer Family Registries (CFR), and the Early Detection Research Network (EDRN) [25]. The technique and means of data and tissue collection vary in each biorepository, although many of these multi-institutional collaborations have been compelled to develop principles for sharing data with other groups due to the pressing need for well annotated tissues that can be re-annotated with experimental data.

The established data elements that are used (and often mandated) in many cancer centers are generally represented by the synoptic template (CAP protocol and checklist and WHO standard) and the NAACCR core elements. In this effort we have demonstrated that the two representations can be pooled to generate a core set of clinical annotations for banked head and neck neoplasm specimens. The collection of information for data elements as been incorporated into the routine hospital workflow so these data sets can be easily developed and maintained. The existing array of elements is currently operating in a database system and we are contemplating means to establish these elements on ISO/IED compliant data standard.

The major distinction between The SPORE in Head and Neck Neoplasm Virtual Biorepository database and most other tissue resources is that all amassed cases have undergone consistent pathology review and the associated clinical data have been carefully collected using a standardized quality-controlled method. A highly effective informatics infrastructure has been designed for the resource that facilitates an efficient governance, standardized data acquisition, and thoroughly standardized case annotation across multiple participating sites. This infrastructure includes an operations manual and a database with common data elements for tissue sample description, clinical follow-up data, and quality assurance procedures.

The web-based query tool allows easy accessibility for IRB- and SRCB-approved collaborators and researchers. Patient confidentiality is top priority. A vibrant campaign for marketing of the SPORE resource, which includes a comprehensive brochure and an interactive web site, is currently underway. We hope that this will encourage institutions and researchers to participate and take advantage of this extremely valuable resource.

## Abbreviations

ADASP: Association of Directors of Anatomic and Surgical Pathology; AJCC: American Joint Committee on Cancer; APLIS: Anatomic Pathology Lab Information System; caBIG: cancer Bioinformatics Grid; CAE: Clinical Annotation Engine; CRIS: Cancer Registry Information System; CAP: College of American Pathologist; CBCTR: Cooperative Breast Cancer Tissue Resource; CDE: Common data element; CPCTR: Cooperative Prostate Cancer Tissue Resource; CPLIS: Clinical Pathology Lab Information System; EDRN: Early Detection Research Network; EMR: Electronic Medical Record; HIPAA: Health Insurance Portability and Accountability Act; HSTB: Health Science Tissue Bank; IATA: International Air Transport Association; IRB: Institutional Review Board; ISO: International Organization for Standardization; MRS: Medical Registry System; NAACCR: North American Association of Central Cancer Registries; NCI: National Cancer Institute; NIH: National Institutes of Health; PCABC: Pennsylvania Cancer Alliance Bioinformatics Consortium; PHI: Protected Health Information; REP: Research Evaluation Panel; SPORE: Specialized Programs of Research Excellence; TBIS: Tissue Bank Information System; UPITT: University of Pittsburgh; UPMC: University of Pittsburgh Medical Center.

## Competing interests

The authors declare that they have no competing interests.

## Authors' contributions

WA and AVP contributed equally to the first draft of this manuscript. AVP and JG is responsible for leading the efforts of developing the requirements for the central database. AVP, JG, WA, HS, TK, MAE, RRS, and JH have contributed in study design, implementation and quality assurance of the database and tool. HS has contributed in the development and implementation of software tools for the data annotation and query engine in the web based interface, and incorporation of other existing standards. Cancer registrars have played an important role in the implementation of cancer registry data standards into the database and contributed in the collection and quality assurance of follow-up and epidemiological data. All authors have reviewed and commented on successive drafts of the manuscript and have provided the first author with approval of the final manuscript.

## Pre-publication history

The pre-publication history for this paper can be accessed here:

http://www.biomedcentral.com/1471-2407/9/396/prepub
